# A novel data augmentation method and a data-driven prediction model for surface flashover at gas–solid interfaces under nanosecond pulses

**DOI:** 10.1038/s41598-026-53820-w

**Published:** 2026-05-27

**Authors:** Chuyu Sun, Haiyang Wang, Gefei Wang, Gang Wu, Jiahui Yin, Tao Huang, Shengchang Ji

**Affiliations:** 1 Northwest Institute of Nuclear Technology, Xi’an, China; 2 Xi’an Jiaotong University, Xi’an, China

**Keywords:** Surface flashover, Data augmentation, Weibull distribution, Feature importance analysis, Energy science and technology, Engineering, Mathematics and computing

## Abstract

Surface flashover at gas–solid interfaces is a critical factor in electromagnetic pulse simulator reliability. To accurately predict flashover events over a wide surface distance range (15–500 mm), this paper develops a machine-learning-based classification model. Three experimental platforms with output ranging from ± 80 kV to ± 2000 kV were constructed, yielding 1245 valid data samples covering various surface distances, voltage polarities, gas pressures, electrode configurations, and voltage waveforms. To address severe class imbalance (flashover proportion > 85%) under certain experimental conditions, a data augmentation method based on the three-parameter Weibull distribution is proposed: non-flashover samples are generated by sampling below a low cumulative probability threshold (*U*_10%_ recommended) after estimating Weibull parameters of flashover voltages, effectively mitigating imbalance and overfitting. Six algorithms including Support Vector Machine (SVM), Multilayer Perceptron (MLP), Random Forest (RF), Gradient Boosting (GB), Extreme Gradient Boosting (XGBoost) and Light Gradient Boosting Machine (LightGBM) are trained with Bayesian hyperparameter optimization. The SVM model achieves the best performance on the test set: F1 score of 0.9111 and AUC of 0.9590. MLP achieves the second-best performance. The tree‑based ensemble methods show slightly lower F1-scores and exhibit a tendency towards overfitting. Feature importance and SHAP analysis are carried out to verify whether the model captures physically consistent mechanisms rather than spurious statistical correlations.

## Introduction

The surface flashover phenomena severely constrain the development of electromagnetic pulse (EMP) simulators towards higher voltage levels^1,2^. If the occurrence of flashover in simulators could be predicted with reasonable accuracy, it would provide significant guidance for the insulation design of higher-voltage devices. However, in EMP simulators, insulation components often face diverse surface insulation environments^3–7^. Surface distance, withstood voltage waveforms, electric field environments and gas pressure all affect the flashover characteristics. Determining the flashover thresholds for each component experimentally would consume substantial manpower and resources.

In recent years, numerous researchers have studied machine learning (ML)-based methods for predicting the flashover voltage of insulators in air. Sima’s research group utilized neural network models to establish predictive relationships between complex environmental conditions and insulator flashover voltage. Through validation with experimental data, they concluded that neural network calculations can essentially reflect the electrical characteristics of insulators^8–10^. Lichun Shu^11^ and Jiangjun Ruan^12^ et al., based on Support Vector Machines (SVM), proposed methods for predicting insulator flashover voltage and rod-plane gap breakdown voltage, respectively, using atmospheric parameters and electric field characteristics as input variables. Abdelhalim Mahdjoubi et al. proposed a fixed support vector set modeling method based on modified Least Squares Support Vector Machines (LS-SVM) for evaluating the flashover performance of outdoor insulators under pollution conditions^13^. Sezavar et al. developed a hybrid model employing Weighted Continuous Wavelet Transform (WCWT) for feature extraction from leakage current data and a Graph Attention Network (GAT) to achieve early prediction of flashover in polluted composite insulators, specifically focusing on critical pre-flashover stages^14^. Research on flashover prediction in SF_6_ is relatively scarce. Chuanjie Lin et al. used tree models to predict the AC flashover voltage of epoxy resin in SF_6_, achieving a model R² coefficient of determination of 0.93^15^.

Despite the existing research progress, investigations specifically focusing on long-distance surface flashover in SF_6_ remain extremely limited by three inherent research challenges. First, acquiring reliable long-distance surface flashover data in SF_6_ poses stringent hardware requirements: an ultra-high voltage pulse source is indispensable, and all auxiliary insulation components within the experimental system must possess significantly higher withstand voltage than the long-distance sample itself, resulting in an extremely bulky, complex, and costly experimental platform. Second, repeated sample replacement involves time-consuming and resource-intensive procedures such as gas recovery and vacuum pumping, leading to substantial consumption of manpower, financial costs, and experimental cycles. Third, for electromagnetic pulse simulation applications, predictive models need to cover flashover behaviors across a wide range of surface distances from short to long gaps; the large span of sample distances imposes great challenges on data collection efficiency, feature learning stability, and model generalization capability. Therefore, it is significant to develop an effective predictive method for long-distance surface flashover in SF_6_. Based on machine learning algorithms, this paper establishes a flashover event prediction model over 15–500 mm distance, enabling the prediction of whether flashover will occur under given insulation conditions.

## Data preparation

### Experimental data acquisition

**Fig. 1:**
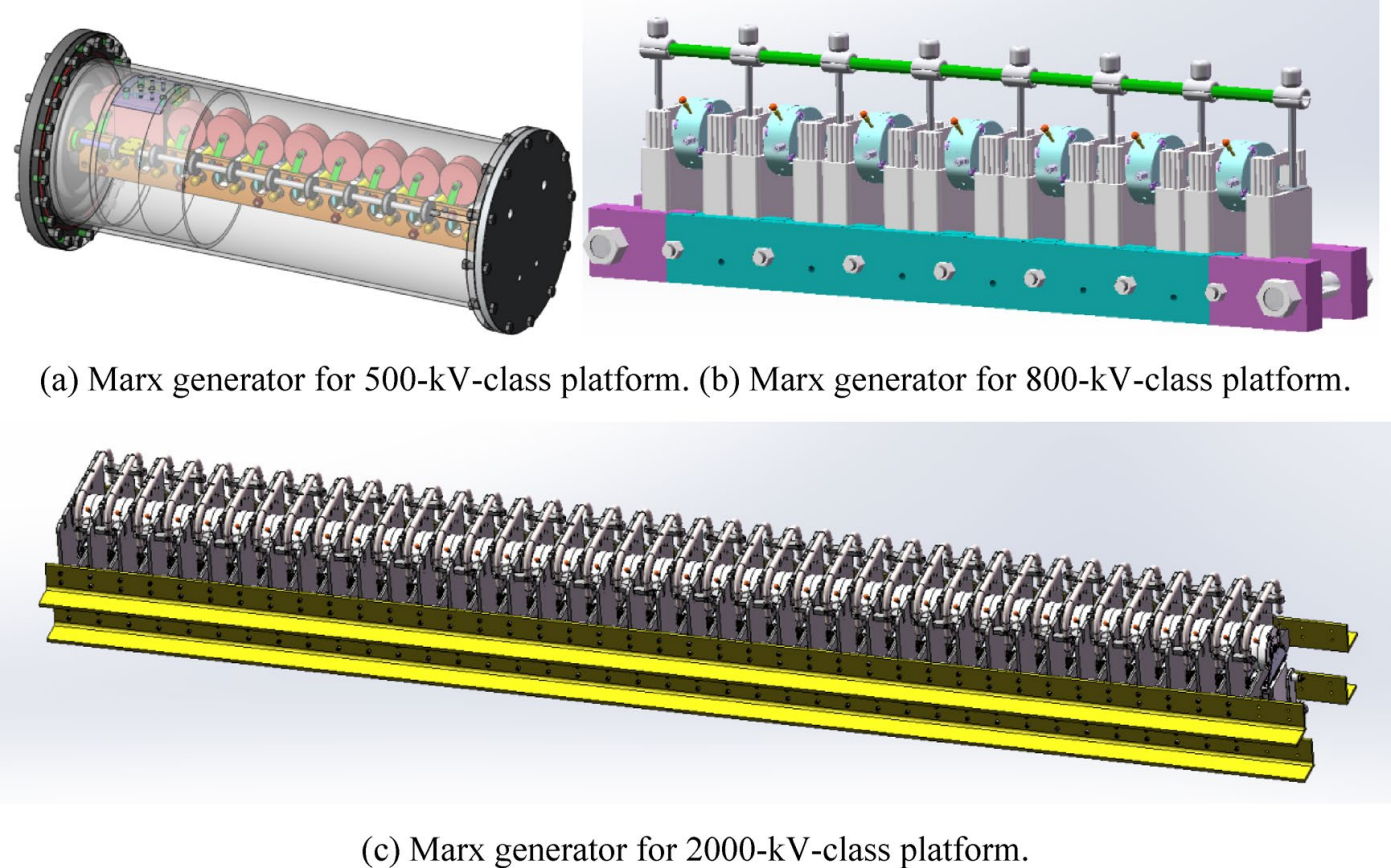
The schematic of Marx generators for three platforms. (**a**) Marx generator for 500-kV-class platform. (**b**) Marx generator for 800-kV-class platform. (**c**) Marx generator for 2000-kV-class platform.

To collect flashover data under various influencing factors, especially within the large-span range of surface distances (15–500 mm), this paper established three experimental platforms rated for 500 kV, 800 kV, and 2000 kV. All three platforms utilize Marx generators as the high-voltage pulse source, as Fig. 1 shows. The pulse voltage output amplitude covers ± 80 kV to ± 2000 kV, meeting the experimental requirements. The pulse source directly discharges to the sample electrodes. The parameter values for the three platforms are listed in Table 1. Nylon is a commonly used material in existing EMP simulators. In this paper, all experimental samples are made of MC nylon, and its typical surface properties are listed in Table 2.

**Table 1:** The parameter values for the three platforms.

	500-kV-class platform	800-kV-class platform	2000-kV-class platform
Output range of voltage amplitude	± 80 kV ~ 500 kV	± 250 kV ~ 800 kV	± 580 kV ~ 2000 kV
2–90% Rise-time of output waveforms	70 ns	110 ns	230 ns

**Table 2:** Typical surface properties of Nylon.

Material	Relative permittivity	Surface resistivity/Ω	Central trap energy level/eV
MC Nylon	3	5.3 × 10^15^	1.81

The flashover experiments were carried out according to the following procedures:

*Step 1* The electrodes and samples were first cleaned with detergent to remove surface oil contamination. Then they were placed in a vacuum drying oven for degassing and drying. Before each test, the surfaces of electrodes and samples were carefully wiped with carbon tetrachloride to ensure cleanliness. The treated electrodes and samples were fixed at the designated positions in the test chamber. All wiping and assembly operations were performed with disposable dust-free PVC gloves to avoid contamination caused by direct contact.

*Step 2* The test chamber was evacuated using a mechanical vacuum pump integrated in the SF_6_ recovery unit to remove residual air. After reaching the preset vacuum level (10^−2^ Pa), high-purity SF_6_ gas (99.9999%) was charged into the chamber to the specified pressure.

*Step 3* Flashover tests were conducted using the step-by-step voltage test method. To suppress the accumulation of space charge, a 1-minute interval was set between each voltage application.

*Step 4* After the test, SF_6_ gas in the chamber was recovered by the SF_6_ recovery unit. The tested samples were removed and replaced with new ones in preparation for the next group of tests. Three samples were tested under each experimental condition.

The experimental conditions for each sample are detailed in Table 3, comprising a total of 1245 data points. Flashover data accounts for 61.7%, while non-flashover data constitutes 38.3%, indicating a relatively balanced overall distribution. However, it should be noted that under specific conditions (highlighted with bold), limited by the lower voltage output limit of the Marx generator, the proportion of flashover samples within the acquired data exceeds 85%, resulting in severe imbalance between positive and negative sample distributions.

**Table 3:** The experimental conditions for each sample.

Surface distance (mm)	Rise-time/half width time(ns)	Electrodes	Gas pressure (MPa)	Voltage polar	Sample number	proportion of flashover samples
15	70/271	Pin–plate	0.1	Positive	22	0.85
				Negative	27	0.44
			0.2	Positive	19	0.78
				Negative	44	0.3
			0.3	Positive	27	0.76
				Negative	46	0.41
15	70/271	Rod–Ring	0.1	Positive	54	0.76
				Negative	33	0.39
			0.2	Positive	46	0.71
				Negative	56	0.27
			0.3	Positive	45	0.73
				Negative	65	0.26
15	70/271	Plane–plane	0.1	Positive	36	0.53
				Negative	43	0.35
			0.2	Positive	39	0.56
				Negative	52	0.27
			0.3	Positive	45	0.51
				Negative	52	0.25
50	110/1960	Plane–plane	0.1	**Positive**	**27**	**0.96**
				Negative	29	0.55
			0.2	**Positive**	**23**	**0.89**
				Negative	19	0.73
			0.3	Positive	28	0.82
				Negative	19	0.68
200	110/1960	Plane–plane	0.1	Positive	42	0.73
				Negative	72	0.39
			0.2	Positive	23	0.60
				Negative	41	0.59
			0.3	Positive	36	0.69
				Negative	19	0.68
300 ~ 500	230/1470	Plane–plane	0.1	**Positive**	**44**	**1**
				**Negative**	**92**	**1**

### Data preprocessing

To address the imbalance issue, this paper adopts a data augmentation method based on the Weibull distribution. The implementation procedure of the proposed method is as follows: first, the Weibull distribution parameters of flashover voltages under imbalanced conditions are estimated, and the goodness of fit is verified by the Kolmogorov–Smirnov (K-S) test; second, non-flashover samples are generated in batches by setting a reasonable threshold; finally, the optimal threshold is selected via sensitivity analysis to supplement the original dataset, thereby completing data augmentation.

**Step 1**

Data augmentation is performed on experimental groups where flashover samples constitute over 85% of the dataset (underlined in Table 2). To eliminate the initial error, a three-parameter Weibull distribution was utilized. the cumulative probability function can be expressed as:

1 $$F\left( V \right)=1 - \exp \left[ { - {{\left( {\frac{{V - \gamma }}{\eta }} \right)}^\beta }} \right]$$

**Fig. 2:**
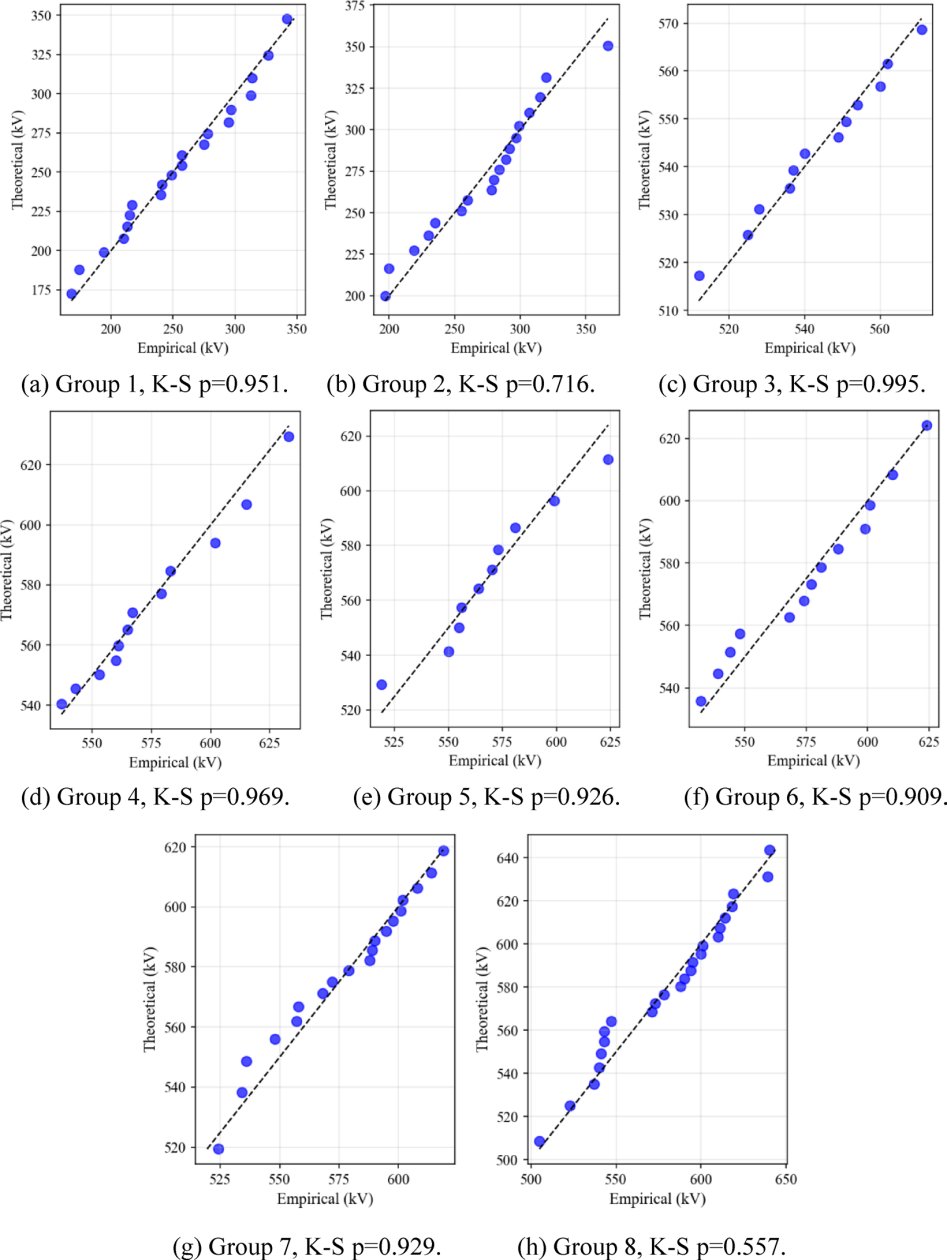
The Q–Q plots of the Weibull distribution. (**a**) Group 1, K-S *p* = 0.951. (**b**) Group 2, K-S *p* = 0.716. (**c**) Group 3, K-S *p* = 0.995. (**d**) Group 4, K-S *p* = 0.969. (**e**) Group 5, K-S *p* = 0.926. (f) Group 6, K-S *p* = 0.909. (**g**) Group 7, K-S *p* = 0.929. (**h**) Group 8, K-S *p* = 0.557.

Where *V* refers to applied voltage; *F*(*V*) is cumulative probability function; *β* represents the shape parameter; *η* represents the scale parameter; *γ* represents the location parameter. The estimation results are determined by Maximum Likelihood Estimation. The Weibull parameter estimates and corresponding K-S test results under each condition are listed in Table 4. For a more intuitive visualization of the fitting performance, the quantile-quantile (Q–Q) plots of the Weibull distribution under each experimental condition are shown in Fig. 2.

**Table 4:** The Weibull parameter estimates and corresponding K-S test results.

Group	Conditions	β	η	γ	63.2% characteristic voltage(kV)	*P*
1	50 mm-positive-0.1 MPa	2.5905	132.19	136.59	268.77	0.9512
2	50 mm-positive-0.2 MPa	3.1261	136.00	152.01	288.01	0.7161
3	300 mm-positive-0.1 MPa	4.3318	67.75	482.20	549.94	0.9952
4	300 mm-negative-0.1 MPa	1.3361	42.75	535.32	578.08	0.9692
5	400 mm-positive-0.1 MPa	2.7369	77.63	499.93	577.56	0.9258
6	400 mm-negative-0.1 MPa	2.1907	64.33	518.83	583.15	0.9090
7	500 mm-positive-0.1 MPa	25.7220	590.57	0.00	590.57	0.9292
8	500 mm-negative-0.1 MPa	4.8320	164.91	429.29	594.21	0.5567

It can be observed that all eight groups exhibit satisfactory goodness of fit, which confirms that the data conform to the Weibull distribution. The shape parameter *β* characterizes the trend of the flashover failure rate with increasing voltage. All values of *β* in this paper are greater than 1, indicating that the failure rate increases with rising voltage. In other words, as voltage increases, more local weak points are activated, leading to a sharp rise in flashover probability. The scale parameter *η* reflects the spread or scale of the distribution for a given shape parameter *β*. When V = *γ* + *η*, the cumulative failure probability is exactly 63.2%. A larger *η* (with *β* unchanged) corresponds to a wider distribution. The location parameter *γ* represents the theoretical threshold voltage below which flashover is statistically impossible. For the 7th group, the estimated *γ* is close to zero, indicating that a non-zero threshold is not supported by the data. This is equivalent to a two-parameter Weibull model and may be attributed to the small sample size and data imbalance, which prevents stable estimation of the three-parameter model.

**Step 2**

The cumulative flashover probabilities of 1, 5, 10, 15, 20, 25, and 30% are selected as thresholds to calculate the corresponding voltage thresholds *U*_th_, respectively. Uniformly spaced sampling is performed within the range from the location parameter γ to *U*_th_, with the number of sampling points consistent with half of the number of the original dataset. For the 7th dataset, a cumulative flashover probability of 0.01% is adopted as the initial sampling threshold, which corresponds to an extremely low flashover probability region far below the actual flashover threshold. The sampled voltage data are used as generated non-flashover samples, which together with other environmental parameters form the input dataset. Using the aforementioned method, a total of 7 data augmentation datasets with different voltage thresholds are generated in this paper.

**Step 3**

To analyze the data augmentation performance under different voltage thresholds, eight SVM classification models are trained with the same workflow based on seven augmented datasets generated in Step 2 and one original dataset. Subsequently, the eight classification models are adopted to predict a specific virtual dataset for performance evaluation. The generation process of the virtual dataset is described as follows.

For the eight severely imbalanced experimental conditions presented in “Data preprocessing” section, input features including electric field, air pressure, gap distance, and time-domain characteristics of voltage waveforms remain consistent with the corresponding experimental parameters. For the voltage amplitude feature, values are sampled at an interval of 5 kV, ranging from 50% to 100% of the Weibull location parameter γ under each experimental condition. For the 7th condition, the voltage amplitude ranges from 0.5*U*_0.01%_ to *U*_0.01%_. From a physical perspective, all samples in the above virtual dataset should correspond to the non-flashover state.

The prediction performance of the eight classification models on the virtual dataset is listed in Table 5. Since the ground truth of all virtual samples is non-flashover, the precision of all eight models is 1.0, leading to the same precision and recall values.

The results indicate that the recall and F1-score of models trained on augmented datasets are greatly improved compared with the model trained on the original dataset. Even the model trained by the *U*_1%_ augmented dataset achieves a far better prediction performance than the original one, which verifies that the proposed data augmentation method can effectively alleviate data imbalance and model overfitting under extreme experimental conditions.

Models trained with the thresholds of *U*_20%_ and *U*_25%_ exhibit the optimal performance, with a recall of 63.35% and an F1-score of 0.7757. When the threshold further increases to *U*_30%_, the model performance degrades significantly, and most samples are misclassified as flashover. This is attributed to the blurred boundary between flashover and non-flashover samples in the *U*_30%_ dataset, which hinders effective feature learning of the model.

Considering that the prediction results of *U*_10%_ and *U*_20%_ are comparable, *U*_10%_ is recommended as the optimal data augmentation threshold in practical engineering applications. That is because insulation design for electromagnetic pulse simulators is safety-critical, where false negatives (missing flashover events) have far more severe consequences than false positives (over-design). Choosing the more conservative *U*_10%_ threshold generates synthetic non-flashover samples at a lower voltage range, which helps the model learn a stricter and safer decision boundary. This setting can effectively avoid missing judgments caused by low-probability flashover events and reduce the risk of under-prediction in actual insulation design.

**Table 5:** The prediction performance of the eight classification models.

Dataset	Accuracy	Precision	Recall	F1-score
Original data	0.1676	1.0000	0.1676	0.2871
Augmented data with thresholds *U*_1%_	0.3835	1.0000	0.3835	0.5544
Augmented data with thresholds *U*_5%_	0.5852	1.0000	0.5852	0.7384
Augmented data with thresholds *U*_10%_	0.6136	1.0000	0.6136	0.7606
Augmented data with thresholds *U*_15%_	0.6278	1.0000	0.6278	0.7714
Augmented data with thresholds *U*_20%_	0.6335	1.0000	0.6335	0.7757
Augmented data with thresholds *U*_25%_	0.6335	1.0000	0.6335	0.7757
Augmented data with thresholds *U*_30%_	0.1591	1.0000	0.1591	0.2745

### Extraction of features on electric field and voltage pulse

The electric field between the electrodes is one of the most critical factors determining the development of the discharge process. In this paper, the electric field distribution along the shortest straight path between the two electrodes is extracted as the feature input parameters for the model, encompassing the following seven aspects:

- Maximum, average, and minimum values of electric field strength;
- Electric field non-uniformity coefficient;
- Maximum and average values of electric field strength gradient;
- Path integral of electric field strength;
- Sum of squared electric field strength and average squared electric field strength;
- Path integral of the squared tangential component of the surface electric field;
- Mean square error of the tangential surface electric field.

Note that aspects 1–4 need to be considered for the electric field magnitude, the tangential component of the electric field along the surface, and the normal component of the electric field along the surface.

Besides the electric field distribution, the applied voltage waveform also affects the flashover voltage. This paper primarily extracts the following features:

- Voltage polarity;
- Voltage amplitude;

Time characteristics, including voltage rise time (*t*_r_), time to half-peak (*t*_50_), and time duration above 89% of the maximum voltage (*t*_89_);

Voltage rise rate (d*u*/d*t*).

Including both electric field and voltage features, a total of 29 feature quantities involving gas pressure, voltage polarity, surface distance, etc., were selected in this study.

Correlations potentially exist among these 29 features, leading to redundancy in the input data which is detrimental to the convergence of the subsequent prediction model. Therefore, this paper employs Pearson correlation analysis to analyze the correlations among these 29 features. This analysis helps screen out features that exhibit strong mutual correlations and features uncorrelated with the output variable. After screening, 22 environmental features and one label feature remained as model inputs, as shown in Table 6.

**Table 6:** The input features.

Feature No.	Class	Features
X1	Surface distance	*D*
X2	Voltage polarity	*Po*
X3	Gas pressure	*P*
X4	Voltage rise time	*t*_*r*_
X5	Voltage rise velocity	d*u/*d*t*
X6, X7	Voltage time features	*t*_*50*_, *t*_*89*_
X8, X9	Maximum of electric field	*E*_max_, *E*_max_y_
X10	Mean squared error of electric field	*E*_std_
X11, X12	Average value of electric field	*E*_ave_, *E*_ave_y_
X13, X14	Nonuniformity of electric field	*E*_f_, *E*_f_y_
X15 ~ X17	Gradient of electric field	*E*_ga_, *E*_ga_x_, *E*_ga_y_
X18	Sum of squares of electric field	*W*_*a*_
X19, X20	Electric field integral of the sum of squares	*W*_*e*_, *W*_*ex*_
X21	Integral of electric field	*V*_90_
X22	Voltage amplitude	*U*_am_
Y	Whether flashover occurs	−1 (unflashover) or 1 (flashover)

It should be clarified that Pearson correlation analysis, feature selection, Weibull data augmentation, as well as the evaluation and optimization of data augmentation thresholds, are offline preprocessing procedures for dataset construction. These steps were completed in a one-time manner based on the entire original dataset before the train–test split, to determine the final input features and sample distribution. In the subsequent formal modeling stage, the normalization strictly followed the principle of dataset separation. Specifically, statistical indicators including mean and standard deviation were calculated only on the training set, and the derived transformation rules were directly applied to the test set.

## Machine learning algorithms and hyperparameter optimization

Six algorithms are adopted for model training in this paper, including the traditional machine learning model Support Vector Machine (SVM), the neural network model Multilayer Perceptron (MLP), as well as tree-based ensemble learning models consisting of Random Forest (RF), Gradient Boosting (GB), Extreme Gradient Boosting (XGBoost) and Light Gradient Boosting Machine (LightGBM). The Bayesian hyperparameter optimization algorithm was utilized to perform hyperparameter tuning for each algorithm to determine reasonable values for the model parameters. To ensure robust model evaluation and hyperparameter selection, we employed stratified ten-fold cross-validation in conjunction with Optuna for hyperparameter optimization. Specifically, the entire dataset was randomly partitioned into 10 folds while preserving the original flashover/non-flashover class distribution in each fold. For each candidate hyperparameter set proposed by Optuna, a model was trained on 9 folds and validated on the remaining 1 fold. This process was repeated for all 10 folds, and the average F1 score across the 10 folds was used as the optimization objective. The Optuna search (conducted for 100 trials) identified the hyperparameters that maximized the cross-validated F1 score. The resulting hyperparameter values are as shown in the Table 7.

**Table 7:** The optimal results of hyperparameter in the six models.

Algorithms	Hyperparameters	Optimization range	Optimal results
SVM	Kernel function	Polynomial function, Radial basis function and Sigmoid function	Radial basis function
	Regularization coefficient	10^−2^~10,000	679.17
	Kernel coefficient	10^−8^~100	6.06
MLP	Number of neurons in the first hidden layer	32 ~ 256	187
	Number of neurons in the second hidden layer	32 ~ 256	197
	Learning rate	10^−4^~10^−2^	0.001
	Regularization coefficient	10^−5^~10^−2^	0.0023
RF	Number of estimators	100 ~ 500	102
	Max depth	5 ~ 30	12
GB	Number of estimators	100 ~ 400	177
	Max depth	3 ~ 10	10
	Learning rate	0.01 ~ 0.3	0.214
LightGBM	Number of estimators	100 ~ 500	337
	Max depth	4 ~ 12	4
	Learning rate	0.01 ~ 0.3	0.186
	Number of leaves	20 ~ 100	33
XGBoost	Number of estimators	100 ~ 500	126
	Max depth	3 ~ 10	5
	Learning rate	0.01 ~ 0.3	0.166

## Training results and model limitations

### Comparison of prediction performance among six models

After the optimal hyperparameters were determined, stratified ten‑fold cross‑validation with fixed optimal hyperparameters was used to obtain the performance of six models. Table 8 shows the mean values and standard deviations of AUC, F1‑score over the ten folds for test set and training set, respectively, which sorted in descending order of average F1-score on test set. The small standard deviations illustrate that the model performance is stable and not sensitive to a particular data split, and the statistical variance is well controlled.

Among them, the SVM model achieves the highest F1-score (0.9111) and AUC (0.9590) on the test set. The MLP model performs comparably, with an F1-score of 0.8973 and AUC of 0.9708. The ensemble tree-based models (XGBoost, RF, GB, LightGBM) yield slightly lower but still competitive results, with F1-scores ranging from 0.8873 to 0.8951.

**Table 8:** The prediction performance of the six models.

Models	Test set		Training set	
	F1 score	AUC	F1 score	AUC
SVM	0.9111 ± 0.0247	0.9590 ± 0.0180	0.9196 ± 0.0032	0. 9708 ± 0.0017
MLP	0.8973 ± 0.0304	0.9474 ± 0.0242	0. 9053 ± 0.0102	0. 9555 ± 0.0057
XGBoost	0.8951 ± 0.0289	0.9541 ± 0.0212	0.9548 ± 0.0014	0.9917 ± 0.0006
RF	0.8891 ± 0.0280	0.9428 ± 0.0227	0.9765 ± 0.0010	0.99272 ± 0.0002
GB	0.8878 ± 0.0323	0.9360 ± 0.0253	0.9771 ± 0.0010	0.9983 ± 0.0001
LightGBM	0.8873 ± 0.0336	0.9487 ± 0.0241	0. 9704 ± 0.0017	0.9965 ± 0.0004

The comparison above leads to the following observations. First, SVM appears to be particularly suitable for this classification task. This may be attributed to the relatively small dataset (1433 samples after augmentation) and the high dimensionality of the feature space (22 features). SVM seeks the optimal separating hyperplane with a kernel trick, which often generalizes well when the number of features is not extremely large relative to the sample size. The small F1-score difference of 0.0085 between the training set and test set further demonstrates that the SVM model has negligible overfitting and strong generalization ability. Second, the MLP, as a representative of neural networks, achieves the second-best performance, suggesting that the relationship between the extracted electric‑field and voltage‑waveform features and the flashover outcome is learnable by a non‑linear feedforward network with appropriate regularization (dropout and weight decay were applied via Bayesian hyperparameter tuning). Third, the tree‑based ensemble methods, despite their strong reputation in tabular data tasks, show slightly lower F1-scores. One possible reason is that these models typically require larger amounts of data to fully exploit their ability to capture complex feature interactions and non‑linearities. With the current dataset size, they may be more prone to overfitting specific splits, even after hyperparameter optimization. It can be seen from Table 8 that all tree-based models achieve high F1-scores on the training set, while their F1-scores on the test set are relatively low, indicating obvious overfitting. Nevertheless, all six models achieve F1-scores above 0.88, indicating that the extracted features are highly informative for predicting surface flashover under nanosecond pulses. Given its superior performance, the SVM model is selected for further analysis.

### Operating point selection and model limitation for practical insulation design

**Fig. 3:**
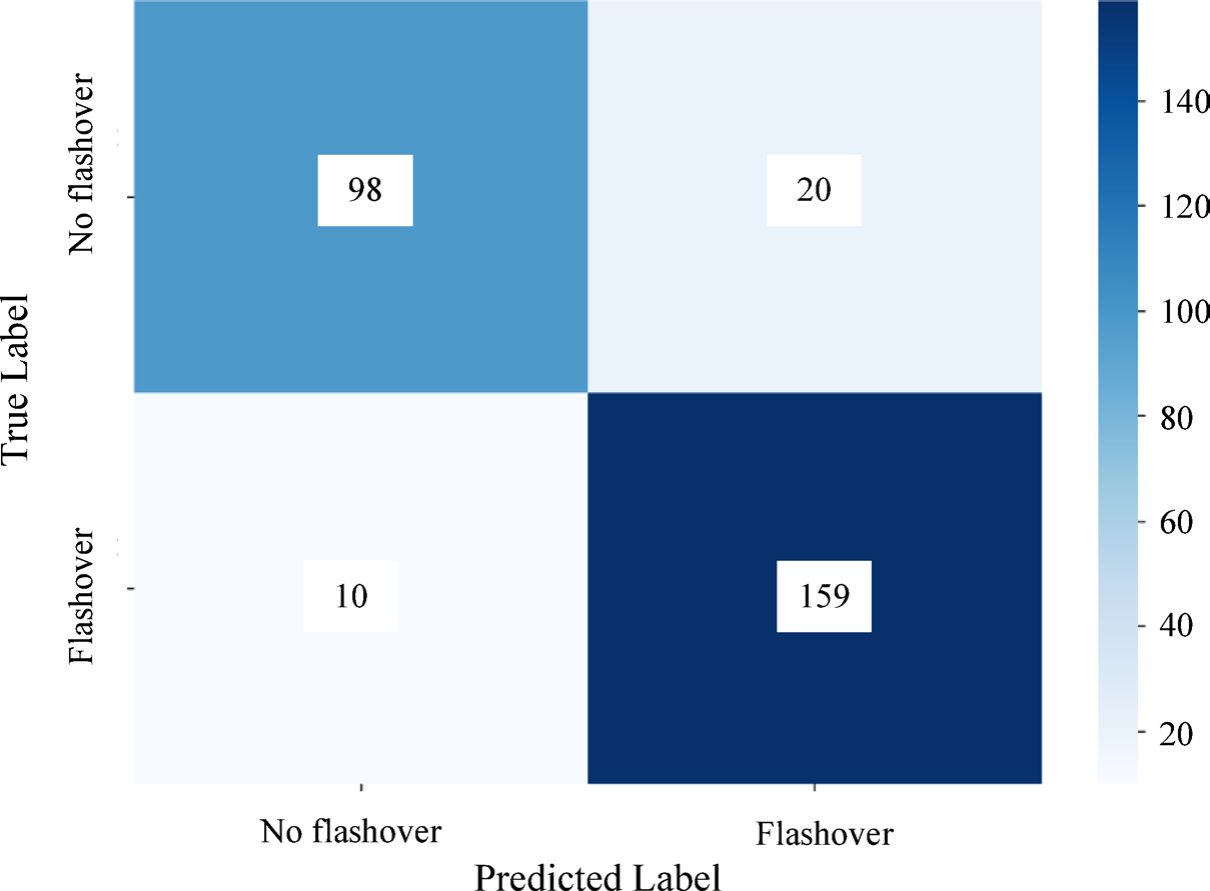
The confusion matrix of SVM model for test set.

The confusion matrix of SVM model for test set is shown in Fig. 3. It can be seen that the model achieves a false negative rate of 5.9% and a false positive rate of 16.9% under the decision threshold of 0.5. In high-voltage insulation design, missing a flashover event (false negative) can have catastrophic consequences, whereas over-design caused by false positives only increases material costs without compromising safety. Therefore, the optimal operating point should prioritize minimizing false negatives.

**Fig. 4:**
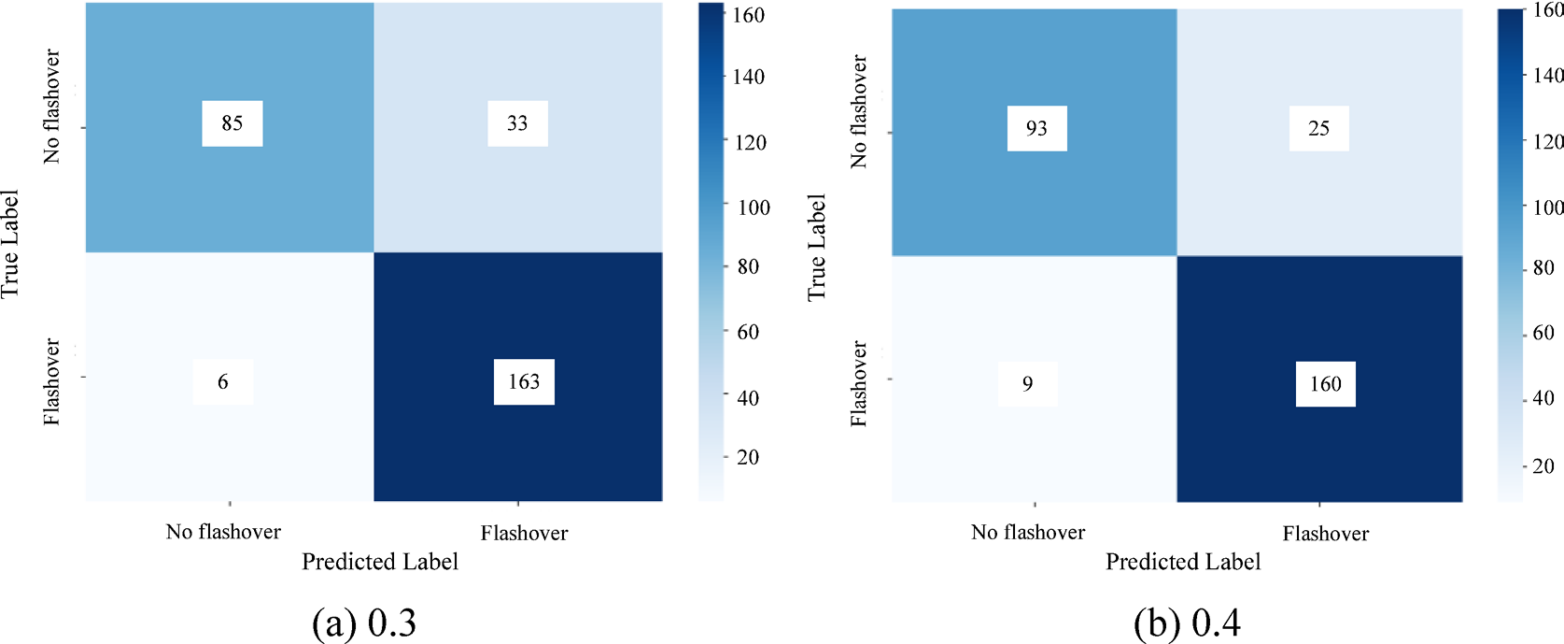
The confusion matrix of SVM model at different decision threshold. (**a**) 0.3, (**b**) 0.4.

Because the SVM model outputs a probability-like score, the decision threshold can be adjusted. Lowering the threshold (e.g., to 0.3) would yield even fewer false negatives (possibly zero) at the cost of more false positives, while raising the threshold would have the opposite effect. Figure 4 illustrates the confusion matrices at decision thresholds of 0.3 and 0.4. As shown, decreasing the threshold improves recall (reduces missed flashover events) but reduces precision (increases false positives). For a given application, the threshold should be chosen based on the relative cost of false positives and false negatives. In safety-critical scenarios, a lower threshold is recommended.

It is important to acknowledge the domain constraints of the current dataset and the resulting predictive model. All experimental data were obtained using the same insulating material (Nylon) under three specific electrode configurations (pin‑plate, rod‑ring, and plane‑plane) in pure SF_6_ atmosphere at pressures of 0.1–0.3 MPa. All validation is performed exclusively under nanosecond‑pulse excitation; thus, the model’s applicability to power‑frequency AC or DC stress has not been verified and should not be inferred. Future work should extend the dataset by including other voltage waveforms, materials, electrode geometries, and gas compositions, and then re-evaluate or fine-tune the model. Within the scope defined above, however, the model provides reliable interpolation predictions that are already useful for guiding the insulation design of existing electromagnetic pulse simulators.

## Model interpretability analysis

**Fig. 5:**
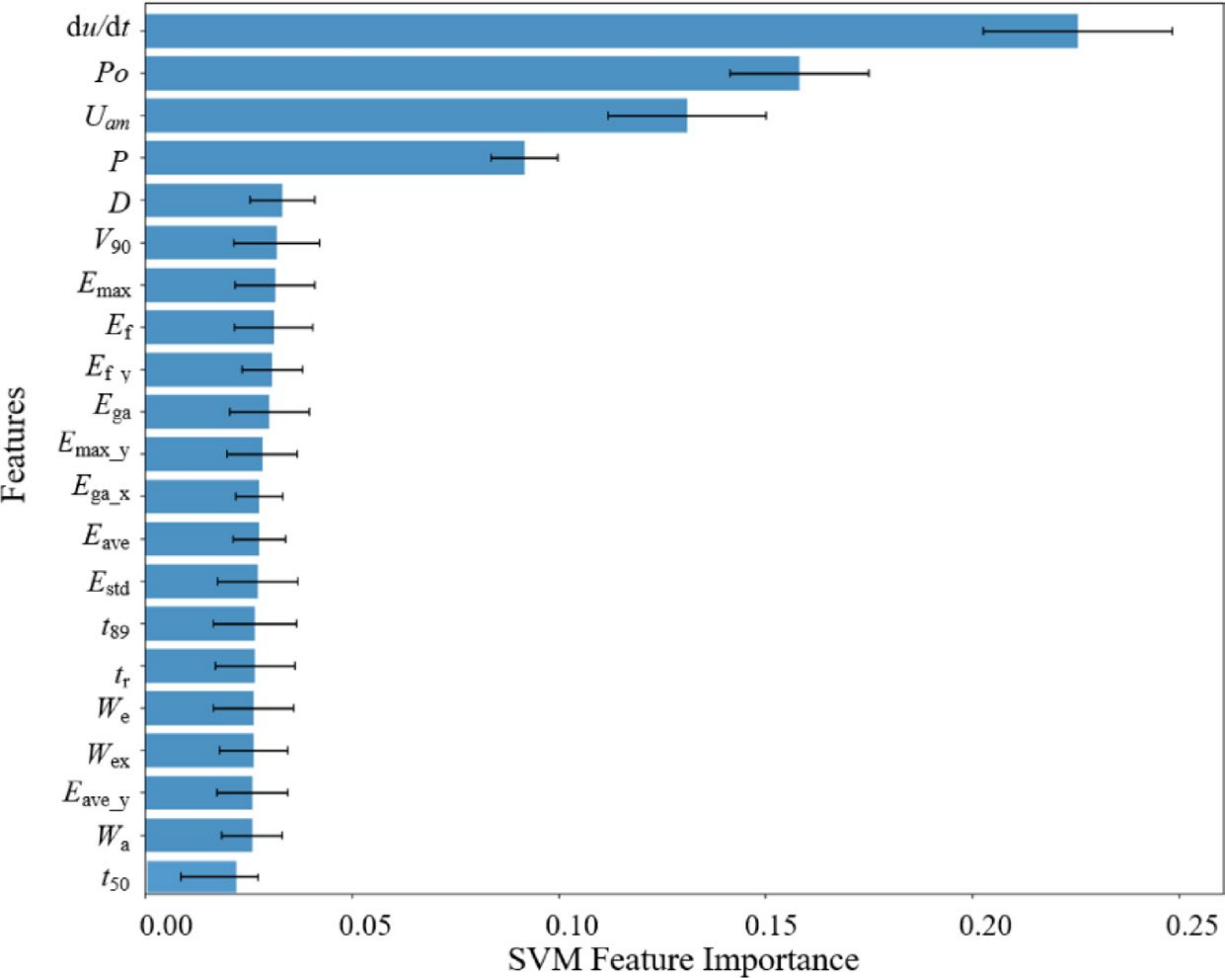
The results of feature importance analysis.

To further verify whether the model captures meaningful underlying patterns consistent with the physical mechanisms of flashover, it is essential to conduct interpretability analysis. This paper conducts a feature importance analysis based on the SVM model, and the results are presented in Fig. 5. It can be observed that the top five dominant features are voltage rise velocity, voltage polarity, voltage amplitude, gas pressure, and surface distance, respectively. Voltage rise velocity (d*u*/d*t*) affects flashover primarily through its control over the available time for streamer or leader initiation and propagation. A high d*u*/d*t* leaves insufficient time for streamer or leader development, thus requiring a higher peak voltage to achieve flashover. Since the space charge under positive polarity strongly enhances the electric field at the streamer head and promotes flashover, the flashover voltage under negative polarity is generally higher than that under positive polarity. In SF_6_, the influence of gas pressure on flashover voltage is rather complex. On one hand, with the increase of gas pressure, the gas density rises and the electron mean free path becomes shorter, so the energy gained by electrons in each collision decreases. A higher electric field is therefore required to reach the ionization threshold, which suppresses the occurrence of flashover. On the other hand, the elevated gas pressure intensifies the space-charge effect and distorts the original electric field, thus reducing the beneficial effect brought by high pressure. Meanwhile, the increase in gas pressure also restrains the thermal expansion process of the stem leader. With the increase of surface distance, flashover characteristics such as the polarity effect and gas pressure dependence change accordingly. This is because, as the surface distance increases from 15 mm to 50 mm and longer, the flashover discharge gradually transitions from being dominated by the streamer mechanism to the leader mechanism^16,17^.

Based on the physical mechanism transition with surface distances, all samples are divided into two groups in this study: short-distance (15 mm) and medium-to-long-distance (50–500 mm). The SHAP values of the top four important features are calculated respectively. Since the distance is kept fixed for the short-distance group, it carries no comparative significance. The relevant results are shown in Table 9. For the short-distance group, polarity has the largest mean absolute SHAP value (0.3452), indicating its dominant influence on flashover prediction. By comparison, in the medium-to-long-distance group, the contribution of polarity drops significantly to 0.1288. Notably, d*u*/d*t* becomes the most influential feature in the medium‑to‑long‑distance group, with a mean absolute SHAP value of 0.2128. Pressure shows a much higher contribution in the short-distance group (0.1095) than in the medium-to-long-distance group (0.0260). In contrast, the voltage amplitude *U*_am_ presents a greater impact on flashover in the medium-to-long-distance group (0.0968) than in the short-distance group (0.0199).

**Table 9:** The SHAP value for different data groups.

Features	Mean absolute SHAP value	
	Distance = 15 mm	Distance ≠ 15 mm
Polarity	0.3452	0.1288
d*u*/d*t*	0.1964	0.2128
Pressure	0.1095	0.0260
*U*_am_	0.0199	0.0968

**Fig. 6:**
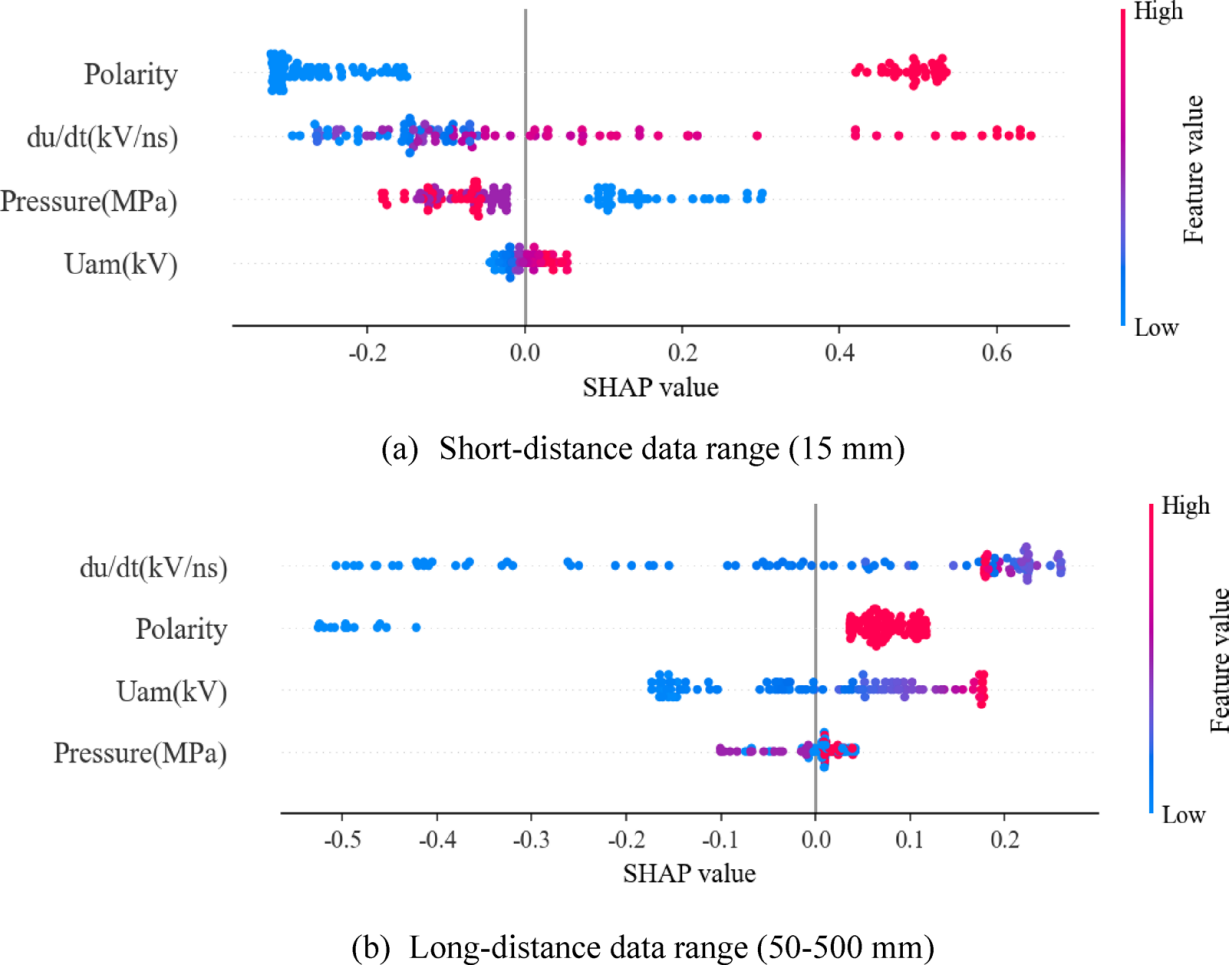
The results of SHAP value.

To better visualize how each feature affects the prediction output both positively and negatively, SHAP distribution plots are provided in Fig. 6. Color represents the feature value: red denotes a high feature value, while blue denotes a low feature value. The horizontal axis corresponds to the SHAP value. A positive SHAP value indicates that the corresponding feature promotes a positive prediction, namely a higher probability of being classified as flashover (1); conversely, a negative SHAP value facilitates the negative prediction, corresponding to the non-flashover state (− 1).

It can be seen from the results that the model’s learning of voltage polarity and voltage amplitude is fully consistent with physical laws. When the voltage polarity is positive (1, high value, red points), the SHAP value is positive; when the voltage polarity is negative (− 1, low value, blue points), the SHAP value is negative. That is, within the established model, positive voltage is more likely to induce flashover than negative voltage. The distinct formation mechanisms of positive and negative streamers result in a pronounced polarity effect in short-distance flashover. As the discharge transits from streamer to leader, the polarity effect gradually weakens. Correspondingly, the discrepancy in SHAP values between positive and negative polarities decreases as the surface distance increases. Meanwhile, high voltage amplitude (red points) yields positive SHAP values, indicating that a larger voltage amplitude increases the predicted probability of flashover. For the voltage rise rate, since the rise time of Marx generator is fixed in this paper, a high amplitude is usually accompanied by a fast rise rate in the present experiment setup, so a high voltage rise rate also presents a positive SHAP value. In terms of gas pressure, high pressure corresponds to negative SHAP values in the short-distance group, which means that a higher pressure suppresses flashover and tends to be predicted as non-flashover. This conclusion is in good agreement with general physical cognition. By contrast, in the medium-to-long-distance group, the influencing direction of gas pressure is less distinct. Some high-pressure samples even show positive SHAP values. This phenomenon is consistent with the experimental results: under medium and long surface distances, the effect of gas pressure on flashover voltage is weak and even shows a slight declining trend, which is closely related to the leader-dominated flashover mechanism at longer distances.

**Fig. 7:**
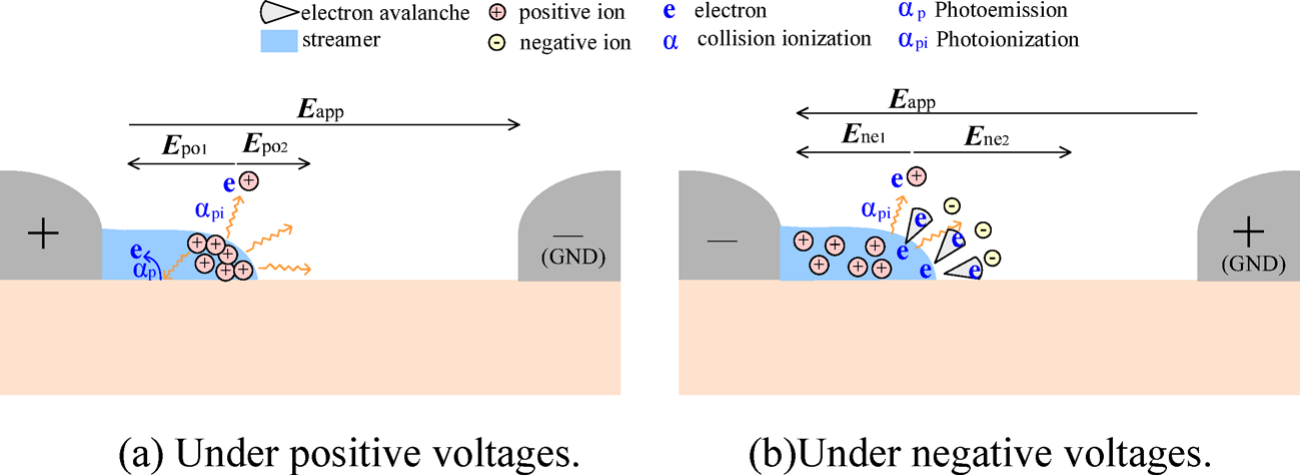
The short-distance flashover. (**a**) Under positive voltages. (**b**)Under negative voltages.

For short distance, streamer is the dominant discharge mechanism, as Fig. 7 shows. For this stage, voltage polarity has a significant influence: under positive voltages, the positive ions concentrate at the streamer head, the electric field generated by these ions enhances the electric field at the head; under negative voltages, the positive charge shields the cathode, and weakens the electric field at the head. This electric field distortion caused by space charge is the main reason for the significantly higher negative polarity flashover voltage compared to positive polarity. In this process, an increase in gas pressure reduces the ionization rate of air, thereby inhibiting the occurrence of surface flashover.

**Fig. 8:**
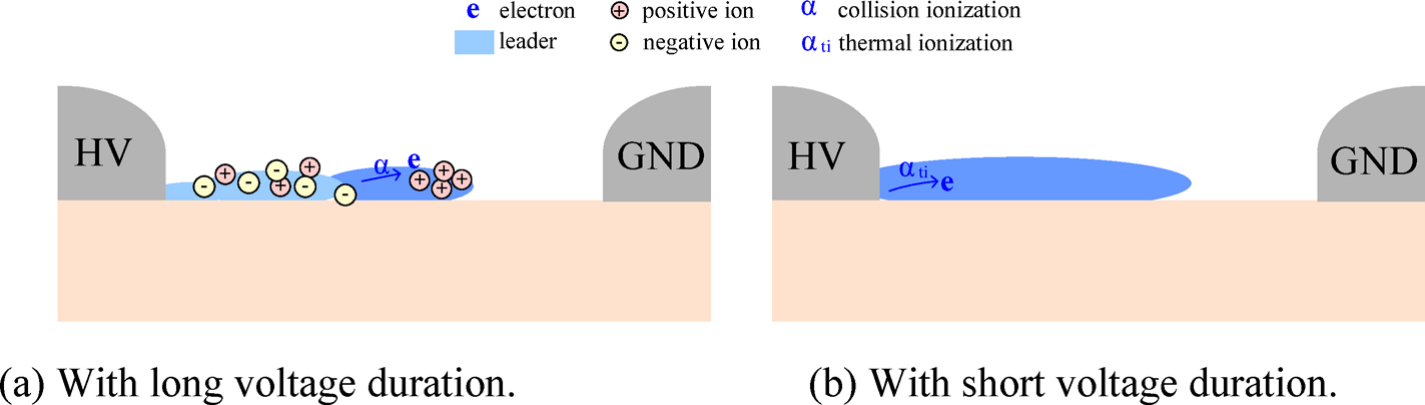
The long-distance flashover. (**a**) With long voltage duration. (**b**) With short voltage duration.

For longer surface distances, the transition from streamer to leader is critical for flashover occurrence, as Fig. 8 shows. In this stage, the influence of voltage polarity diminishes, and voltage application time becomes the core influencing factor, causing the significant difference between front-flashover voltage and tail-flashover voltage. When voltage application time is sufficiently long, flashover development is dominated by the precursor leader mechanism, focusing on space charge migration at the head of the leader. At this time, the increase in gas pressure intensifies the accumulation of space charge at the discharge head and promotes the formation of precursor leaders. When voltage application time is extremely short, flashover mainly occurs via the stem leader mode, focusing on continuous heating and radial expansion at the root of the streamer channel. This excellent consistency between the quantitative model interpretation and the qualitative physical mechanism analysis strongly demonstrates that the proposed model does not merely fit statistical data, but effectively captures the essential physical laws of surface flashover evolution.

## Conclusion

Based on a pulsed flashover experimental platform from ± 80 kV to ± 2000 kV, this study acquired flashover data under multiple factors over surface distances of 15–500 mm. After feature engineering and machine learning modeling, the main conclusions are as follows:

1. To address severe class imbalance (flashover proportion > 85%) under 8 specific conditions, a Weibull-distribution-based method for generating non-flashover samples is proposed. Goodness-of-fit of Weibull distribution is verified by the Kolmogorov–Smirnov test. Based on engineering considerations and sensitivity analysis results, *U*_10%_ is recommended as the practical sampling threshold. At this threshold, this augmentation method increases the recall on a virtual non-flashover test set from 16.8% to 61.4%, significantly mitigating overfitting.
2. Among six algorithms, the SVM model achieves the best performance on the test set, with an F1 score of 0.9111, an AUC of 0.9590. the MLP achieves the second-best performance. The tree‑based ensemble methods show slightly lower F1-scores.
3. The feature importance analysis and SHAP analysis further validates the physical rationality of the model, showing clear consistency with prior mechanism insights: For short-distance flashover, discharge is dominated by streamer discharge, and the most critical factor affecting prediction accuracy is voltage polarity. For long-distance flashover, discharge is dominated by leader discharge, and the most critical factor affecting prediction accuracy is voltage rise rate and duration. This confirms that the model captures the essential physical trends rather than pure statistical fitting.

## Data Availability

The data used to support the findings of this study are available from the corresponding author upon reasonable request.
